# Acute tubulointerstitial nephritis associated with antineutrophil cytoplasmic antibody following cimetidine treatment: a case report

**DOI:** 10.1186/s12882-021-02502-y

**Published:** 2021-08-30

**Authors:** Keita Morimoto, Go Kanzaki, Takahito Niikura, Kentaro Koike, Nanae Matsuo, Yukio Maruyama, Nobuo Tsuboi, Takashi Yokoo

**Affiliations:** Division of Nephrology and Hypertension, Department of Internal Medicine, The Jikei University School of Medicine, Minato-ku, 105-8461 Tokyo, Japan

**Keywords:** Acute tubulointerstitial nephritis, Antineutrophil cytoplasmic antibody-associated vasculitis, Myeloperoxidase-antineutrophil cytoplasmic antibody, Cimetidin

## Abstract

**Background:**

Antineutrophil cytoplasmic antibody (ANCA)-associated vasculitis usually induces rapidly progressive glomerulonephritis, including pauci-immune necrotizing crescentic glomerulonephritis. Acute tubulointerstitial nephritis (ATIN), which is often drug-induced, is a frequent cause of kidney injury. However, ATIN associated with ANCA without any glomerular lesions has been rarely reported, and drug-induced ATIN associated with ANCA is not well recognized. Here we present a case of an older woman with ATIN associated with myeloperoxidase-ANCA (MPO-ANCA) following cimetidine treatment.

**Case presentation:**

A 70-year-old woman was admitted to our hospital due to acute kidney injury and mild proteinuria. She had a one-year history of chronic thyroiditis and dyslipidemia, for which she was taking levothyroxine sodium and atorvastatin, respectively. Two weeks before admission she had started cimetidine, methylmethionine sulfonium chloride, and itopride hydrochloride for gastric discomfort persistent since a month. She had experienced fatigue for two weeks and later appetite loss. The patient demonstrated a positive titer for MPO-ANCA (192 IU/mL) and a positive drug-induced lymphocyte stimulation test for cimetidine. She underwent two kidney biopsies that revealed ATIN without any glomerular lesions. Despite discontinuation of cimetidine on admission, renal injury continued with the presence of high MPO-ANCA titer. Oral steroid treatment was closely related with the recovery of her renal function and disappearance of MPO-ANCA.

**Conclusions:**

In this case, ATIN presented as sustained renal insufficiency and high MPO-ANCA titer despite withdrawal of cimetidine. Therefore, we reason that the development of ANCA-associated ATIN was caused by cimetidine. Serologic follow-up with measurement of MPO-ANCA titers and renal biopsy are recommended when the clinical history is inconsistent with the relatively benign course of drug-induced ATIN.

## Background

Antineutrophil cytoplasmic antibodies (ANCAs) are autoantibodies that use neutrophil cytoplasmic granules and lysosomes as corresponding antigens. ANCAs activate neutrophils by inducing a neutrophil-related cell death [1].

MPO-ANCA-associated vasculitis typically leads to rapidly progressive glomerulonephritis with pauci-immune crescent formation [2]. Pauci-immune crescentic glomerulonephritis is usually accompanied by a certain degree of tubulointerstitial lesions. As the tubulointerstitial injury is thought to occur secondary to the glomerular injury, the current histopathologic classification of ANCA-associated glomerulonephritis has been focused on the glomerular lesions [3, 4].

Rare cases of acute tubulointerstitial nephritis (ATIN) associated with ANCA have been reported, presenting as pure interstitial nephritis without any glomerular lesions [5]. These cases have mostly been related to drugs or systemic disease [6–8]. Although certain drugs, such as propylthiouracil, may play a role in the pathogenesis of AAV [9, 10], the relationship between most drugs and ATIN associated with ANCA is tentative and the pathological mechanisms are unknown. Most cases of drug-induced AAV improve after withdrawal of the suspected drug, but in severe cases, steroids may be required [11]. It is, therefore, difficult to distinguish between drug-induced AAV and primary AAV based on clinical symptoms, laboratory markers, and pathological findings [11].

Cimetidine, a histamine type-2 receptor antagonist, is a major drug indicated for peptic ulcer and gastroesophageal reflux disease. Cimetidine often causes acute kidney injury as a side effect, but its association with AAV is unknown. Here, we describe a case of an older woman presenting with ATIN and a high titer of MPO-ANCA while taking cimetidine, who underwent repeated renal biopsy to assess the persistence of renal insufficiency after drug cessation.

## Case presentation

A 70-year-old Japanese woman with a two-week history of fatigue and subsequent appetite loss was admitted to our hospital due to acute kidney injury (AKI). She had a one-year history of chronic thyroiditis and dyslipidemia, for which she was taking levothyroxine sodium and atorvastatin, respectively. One month before admission, she suffered from gastric discomfort, and thus she took cimetidine, methylmethionine sulfonium chloride, and itopride hydrochloride for two weeks prior to admission.

On admission, she had no upper respiratory symptoms or arthralgia. Her body temperature was 36.7 °C, and blood pressure was 123/77 mm Hg. Physical examinations of the heart, lungs, abdomen, and nervous systems were unremarkable. The right costovertebral angle was tender. There was no pitting edema or palpable purpura of the lower extremities. The suspected drugs, including cimetidine, were discontinued.

Laboratory studies revealed white blood cell count of 12,700/µL (neutrophils, 11,600/µL; lymphocytes, 600/µL; monocytes, 500/µL; eosinophils, 200/µL). Her serum creatinine (s-Cr) concentration was high at 5.81 mg/dL as compared to 0.5 mg/dL at one year before the admission. The level of C-reactive protein was also increased (14.89 mg/dL). Her antinuclear antibody and anti-Sjögren antigens A and B were negative. The titers of MPO-ANCA and proteinase 3 ANCA were 192 U/mL and 9.2 U/mL, respectively. The urinalysis showed urinary pH 6.5, mild proteinuria (1+) with mild occult blood (1+), and no urinary glucose. Moreover, the analysis of the sediment showed 1–4 red blood cells and 50–99 white blood cells per high power field (×400). No eosinophils were found in the urine. In addition, the analysis of urinary excretion of N-acetyl-beta-D-glucosaminidase and the beta-2 microglobulin revealed their concentrations to be 13.8 IU/L and 546 µg/mL, respectively. Urinary protein excretion was 1.32 g/24 h and 24-hour creatinine clearance was 14 mL/min. Blood and urine cultures were negative. Computed tomography showed enlarged kidneys but no signs of urinary obstruction and no abnormal shadow in the lung field. A drug-induced lymphocyte stimulation test of peripheral blood was positive for cimetidine (602 cpm, stimulation index 289 %).

**Fig. 1:**
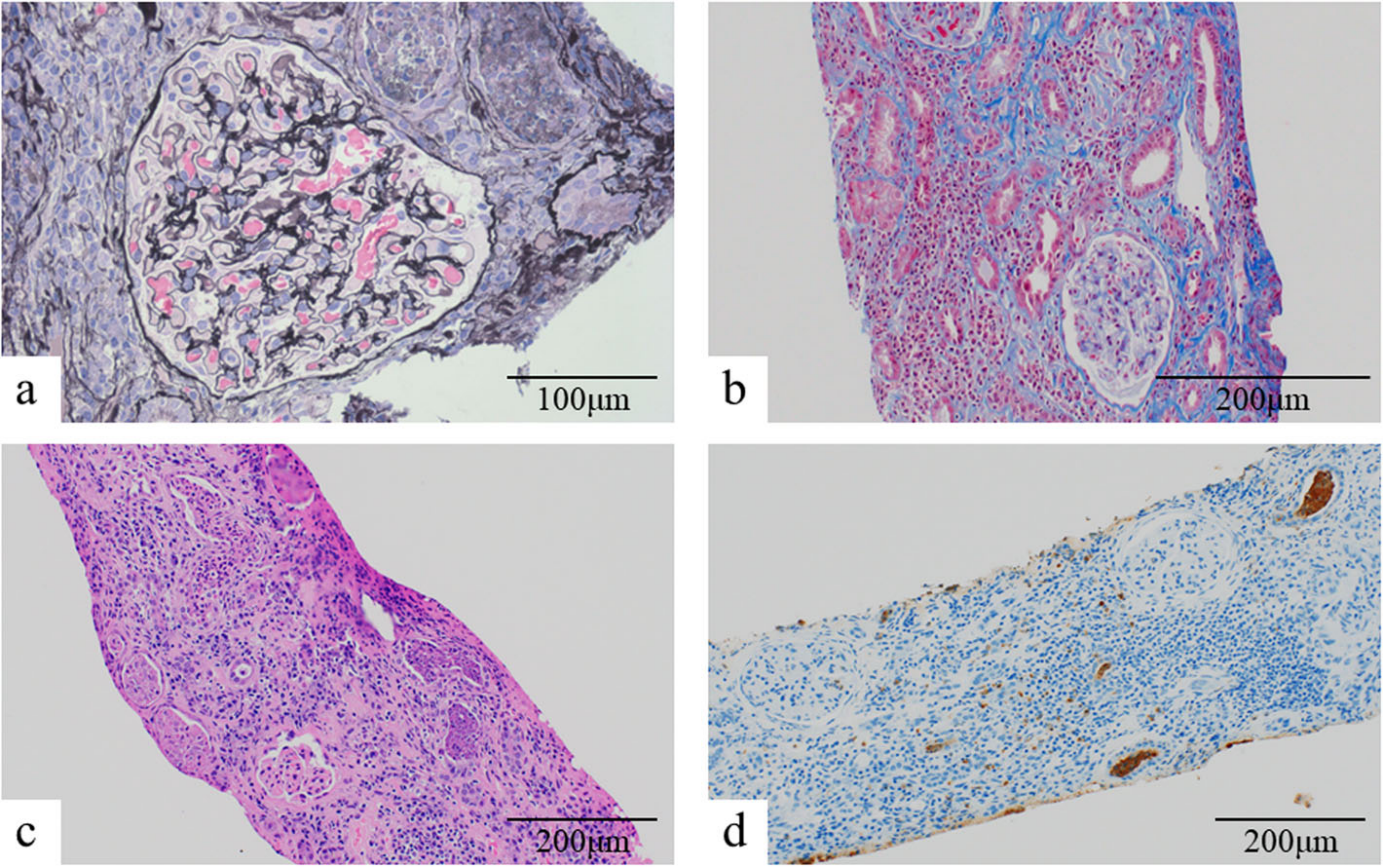
The pathological findings of the first renal biopsy. **a **Light microscopic examination showed 11 intact glomeruli (PAM stain, ×400). **b**, **c** A severe and diffuse infiltration of inflammatory cells composed of mainly plasma cells, lymphocytes, and neutrophils was observed in the interstitial compartment, together with mild to moderate fibrosis of the interstitium. Leukocyte casts, mainly neutrophils, were observed in the renal tubules (**b**, Masson’s trichrome stain, ×100, **c** hematoxylin-eosin stain, ×200). **d** Immunochemistry showed MPO-positive cells in the interstitial compartment (**d** MPO immunohistochemical staining, ×200). MPO, myeloperoxidase

In further evaluation of the cause of AKI, a renal biopsy was performed five days after admission. Light microscopy (Olympus BX-53 microscope, Olympus Optical Co. Ltd.) revealed 11 glomeruli, all of which were intact (Fig. 1a). The glomerular basement membrane showed no thickening or irregularity. Approximately 90 % of the renal cortex showed signs of tubulointerstitial injury (Fig. 1b). The interstitial compartment was severely and diffusely infiltrated by plasma cells, lymphocytes, monocytes, neutrophils, and eosinophils, accompanied by mild to moderate fibrosis (Fig. 1c). Peritubular capillaritis and leukocyte casts with prominent neutrophil infiltration were observed in the interstitium (Fig. 1d). These photomicrographs were captured using an Olympus DP74 digital camera. Immunofluorescent and electron microscopic examination did not show any deposits of immunoglobulins or complement components. Based on renal biopsy findings, we diagnosed the condition as ATIN due to drugs, acute pyelonephritis, and/or AAV.

We started treatment with intravenous ampicillin-sulbactam for acute pyelonephritis. Due to the urinary tract infection and lack of characteristic AAV, corticosteroid administration was not initiated after the first biopsy and the patient was followed up. The patient’s renal function improved slightly with antimicrobial therapy and cessation of cimetidine, but s-Cr was 1.69 mg/dL and MPO-ANCA titer was 121 U/mL on day 31 after admission.

**Fig. 2:**
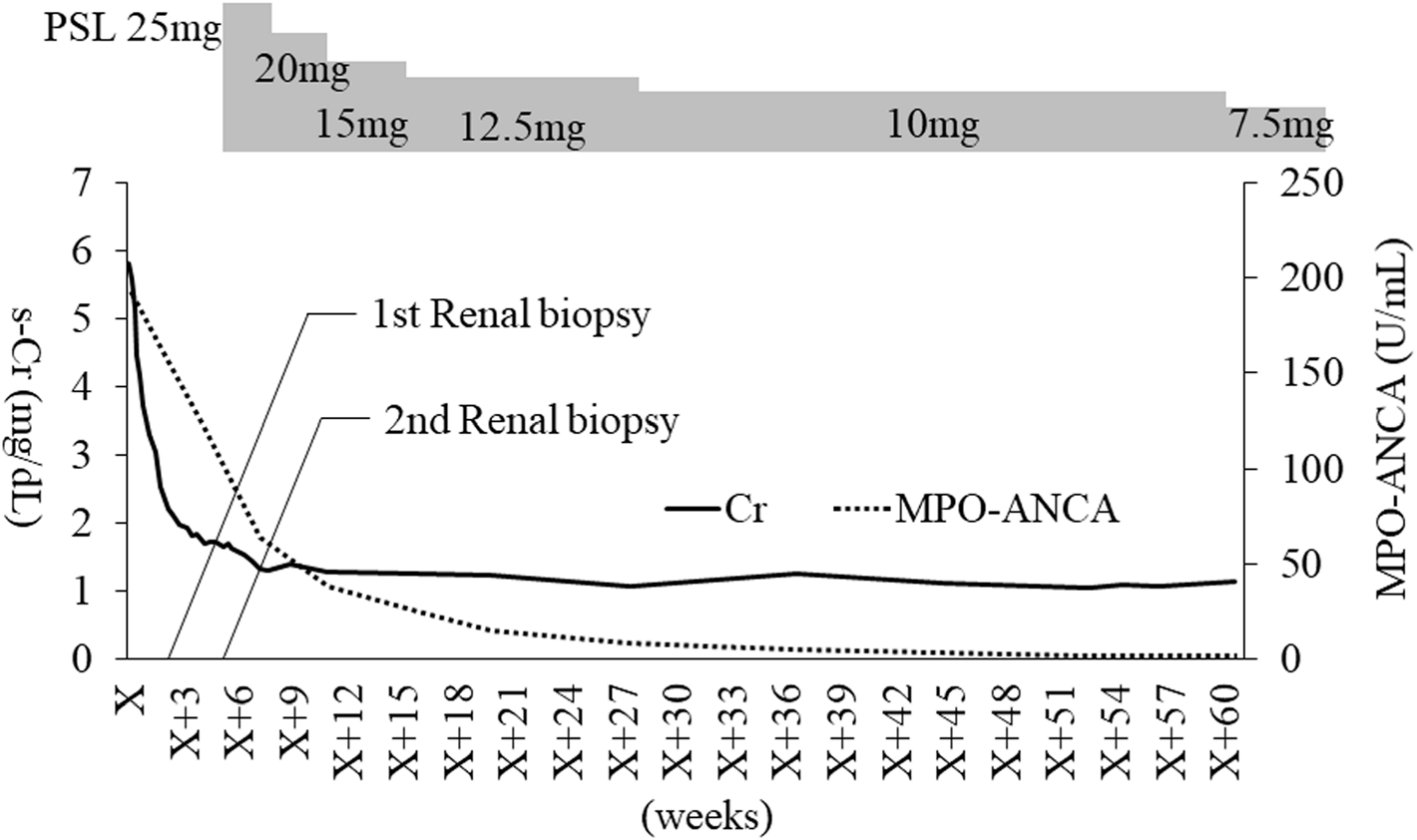
Clinical course and the sequential determination of MPO-ANCA. The first renal biopsy was performed five days after admission, and the second renal biopsy was performed 31 days after admission. On day 40 after admission, the patient received an initial 25 mg/day (0.6 mg/kg) PSL dose orally. As a result, her s-Cr decreased gradually. One year later, she was receiving 7.5 mg/day PSL dose, and her s-Cr has been stable around the level of 1.1 mg/dL. PSL, prednisolone; s-Cr, serum creatinine concentration; MPO-ANCA, myeloperoxidase-antineutrophil cytoplasmic antibody

**Fig. 3:**
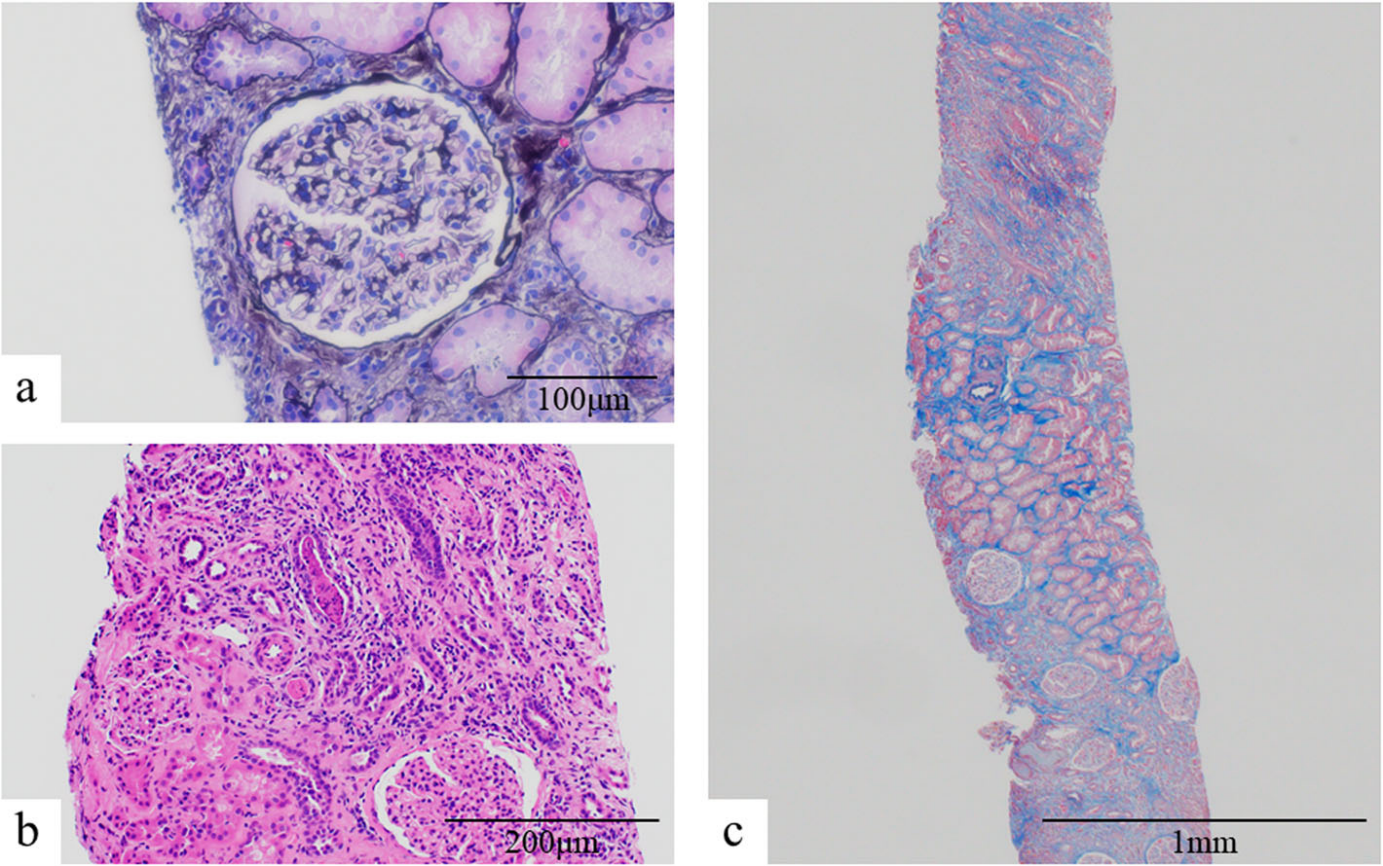
The pathological findings of the second renal biopsy. **a **Light microscopic examination showed 30 glomeruli, of which three were globally sclerotic (PAM stain, ×400). **b**, **c **Light microscopic examination showed that the interstitial compartment was focally infiltrated with inflammatory cells composed of mainly plasma cells, lymphocytes, and a slight mixture of neutrophils. The extent of tubulointerstitial injury was ~ 50 % of the renal cortex (**b **hematoxylin-eosin stain, ×200; **c **Masson’s trichrome stain, ×40)

Suspecting that MPO-ANCA-associated vasculitis was the most probable explanation of the prolonged renal impairment, we performed a second renal biopsy on day 31 after admission (Fig. 2). Light microscopic assessment of the biopsy specimen showed 30 glomeruli, of which only three were globally sclerotic and no other glomerular lesions were observed. The interstitial compartment was focally infiltrated with inflammatory cells, (mainly plasma cells, lymphocytes, and a slight mixture of neutrophils). Approximately 50 % of the renal cortex showed signs of tubulointerstitial injury (Fig. 3a, b, c). No arteritis or arteriolitis was observed. Immunohistochemical analysis showed no expression of IgG, IgM, IgA, C3, and C1q. There were no subepithelial, subendothelial, mesangial, or tubular electron-dense deposits, as observed by electron microscopy. The second renal biopsy showed that ATIN persisted although it had improved compared with the first renal biopsy findings. We diagnosed ATIN associated with MPO-ANCA because renal injury persisted with the presence of MPO-ANCA despite discontinuation of cimetidine and antibiotic therapy.

At 40 days after admission, our patient was started on 25 mg (0.6 mg/kg) oral prednisolone per day (Fig. 2), with gradual tapering to 7.5 mg/day over a year. Improvement in renal function was shown by s-Cr dropping to 1.1 mg/dL and MPO-ANCA seroconversion changing from positive to negative after one year.

## Discussion and conclusions

We reported a case of MPO-ANCA-positive ATIN suspected to be caused by cimetidine. Although side-effects of cimetidine and urinary tract infection were suspected as causes of AKI on admission, ATIN related to AAV was considered based on two renal biopsy findings and the MPO-ANCA antibody titer.

Glomerular necrosis and crescents are the most common and most distinctive histopathologic features of ANCA-associated glomerulonephritis [2]. However, in a study of 232 renal biopsy specimens from ANCA-positive patients, light microscopy revealed no glomerular lesions or only tubulointerstitial inflammation in ~ 1 % cases [3]. These patients may have had focal ANCA glomerulonephritis that was not detected in the kidney biopsy specimens; ANCA disease with only tubulointerstitial nephritis and no glomerulonephritis was suggested by Lockwood [4]. In addition, a few patients with AAV have been reported to have only tubulointerstitial nephritis with apparently no glomerular lesions [6–8].

In our case, MPO-ANCA was strongly positive (192 U/mL) at the time of admission, and ATIN without glomerular lesions was observed in both biopsies. Although ATIN has a variety of causes, including drugs, systemic infections, and autoimmune diseases, the high MPO-ANCA titer and renal damage persisted despite antibiotic treatment for urinary infection and discontinuation of the suspected drug. Therefore, we concluded that the cause of AKI in this case was ATIN related to MPO-AAV.

There have been several reports regarding ATIN associated with ANCA that were linked to drugs or to confirmed or suspected systemic vasculitis [6–8]. To the best of our knowledge, there have been only eight reports of drug-induced ANCA-associated ATIN (Table 1) [12–19]. In most cases, renal function improved. In only one case, methylprednisolone pulse therapy was effective for a limited time, but renal function and ANCA titers worsened again, eventually leading to death from sepsis [13]. Kitahara et al. reported a case of cimetidine-induced ATIN, which was associated with ANCA [18]; ATIN developed in a 63-year-old man who had been taking cimetidine for the treatment of gastric ulcer. They concluded that cimetidine was responsible for the development of both ATIN and ANCA because ANCA became undetectable immediately after cessation of cimetidine. Ueda et al. reported a case of cimetidine-induced tubulointerstitial nephritis with both MPO-ANCA and PR3-ANCA [19]. They found that withdrawal of cimetidine was also associated with rapid disappearance of ANCA. In contrast, the MPO-ANCA titer and s-Cr concentration in our case decreased gradually over one year after treatment with oral prednisolone. These findings suggest that cimetidine may induce MPO-ANCA-associated ATIN rather than ATIN and MPO-ANCA independently.

**Table 1:** Reports of drug-induced ATIN associated with ANCA

Authors	Age (years)	Sex	Drug	ANCA	Treatment	Renal function	References
Dolman et al.	32	F	Propylthiouracil	MPO and PR3	ND	Improved	[12]
Dolman et al.	45	F	Propylthiouracil	MPO	ND	Improved	
Dolman et al.	37	F	Propylthiouracil	MPO and PR3	ND	Improved	
Kitahara et al.	39	F	Propylthiouracil	MPO and PR3	ND	Improved	[13]
Nishimura et al.	54	M	Propylthiouracil	MPO	PSL	Improved	[14]
Shin et al.	81	M	Ciprofloxacin	MPO	ND	ND	[15]
Sakai et al.	83	M	Indomethacin	MPO	mPSL	Death	[16]
Feriozzi et al.	65	M	Cephotaxime	MPO	cessation	Improved	[17]
Kitahara et al.	63	M	Cimetidine	MPO	cessation	Improved	[18]
Ueda et al.	75	M	Cimetidine	MPO and PR3	cessation	Improved	[19]
This case	70	F	Cimetidine	MPO	PSL	Improved	

*ND *not described

The exact pathogenetic mechanism of ATIN in ANCA-associated nephritis remains unknown; various hypotheses have been considered for the pathogenesis, including the following: (i) direct spillover of glomerular capillaritis, (ii) peritubular capillaritis, (iii) direct spillover of vasculitis in vessels larger than arterioles, (iv) extravascular granuloma formation, and (v) overwork of residual tubules [20].We found infiltration of neutrophils and mononuclear cells in the peritubular capillary lumens and in the surrounding interstitium, which suggests that ATIN in this case was due to peritubular capillaritis. The renal biopsy results showed no evidence of glomerular capillaritis or arteritis but revealed mainly peritubular capillaritis with prominent neutrophil infiltration of the interstitium. Based on these pathological findings, ATIN associated with MPO-ANCA in our case was presumed to have resulted from peritubular capillaritis.

Patients receiving cimetidine may have a mild increase in s-Cr. ATIN rarely occurs and s-Cr levels quickly return to normal after cimetidine cessation. Cimetidine has been reported to enhance cell-mediated immunity, inhibit antigen-specific suppressor T-lymphocyte function, and increase serum levels of immunoglobulins [21]. However, the detailed mechanism by which cimetidine increases s-Cr is not well understood, and the causal relationship with ANCA is unknown. Ueda et al. described the cimetidine-induced ATIN associated with MPO-ANCA and PR3-ANCA [19]. They indicated that these immunomodulatory actions could result in dysregulation of the immune system, thereby leading to polyclonal activation of B cells and even to the generation of harmful autoantibodies in cimetidine-induced tubulointerstitial nephritis with ANCA.

Considering that no previous tests for vasculitis or ANCA titers had been performed, the causal relationship with cimetidine could not be reliably established in our case, but based on the clinical course at a family doctor, it was unlikely that renal dysfunction or vasculitis occurred previously. In addition, there was one report of drug-induced AAV caused by atorvastatin [22]. The case involved 45-year-old man with pain in both legs, numbness in his limbs, and hearing loss. He was diagnosed with drug-induced AAV, based on mononeuritis multiplex, sensorineural hearing loss, significantly increased MPO-ANCA titers, and statin-induced distal myopathy. Unlike him, no muscular or neurological symptoms were observed in our case. In addition, she continued to take atorvastatin both before and after the treatment, so it is unlikely that atorvastatin was the cause of drug-induced AAV. Therefore, it is reasonable that our case was indeed ATIN associated with MPO-ANCA following cimetidine treatment. Further cases need to be analyzed to unravel the pathogenesis of drug-induced AAV.

In conclusion, we have reported a case of ATIN associated with ANCA following cimetidine treatment. When drug-induced renal disorders persist even after withdrawal of the suspected drug, it is necessary to consider further diagnostic workup including renal biopsy and the measurement of ANCA titer.

## Data Availability

Not applicable.

## Abbreviations

ANCA: Antineutrophil cytoplasmic antibody
AAV: Antineutrophil cytoplasmic antibody-associated vasculitis
MPO: Myeloperoxidase
ATIN: Acute tubulointerstitial nephritis
AKI: Acute kidney injury
s-Cr: Serum creatinine
